# Biomimetic Intrafibrillar Mineralization of Native Tendon for Soft–Hard Interface Integration by Infiltration of Amorphous Calcium Phosphate Precursors

**DOI:** 10.1002/advs.202304216

**Published:** 2023-10-23

**Authors:** Yangwu Chen, Yuxiang Zhang, Xiaoyi Chen, Jiayun Huang, Bo Zhou, Tao Zhang, Wei Yin, Cailian Fang, Zi Yin, Haihua Pan, Xiongfeng Li, Weiliang Shen, Xiao Chen

**Affiliations:** ^1^ Dr. Li Dak Sum & Yip Yio Chin Center for Stem Cells and Regenerative Medicine and Department of Orthopedic Surgery of The Second Affiliated Hospital Zhejiang University School of Medicine Hangzhou 310058 P. R. China; ^2^ Department of Plastic Surgery Sir Run Run Shaw Hospital, School of Medicine, Zhejiang University Hangzhou 310000 P. R. China; ^3^ Key Laboratory of Tissue Engineering and Regenerative Medicine of Zhejiang Province Zhejiang University Hangzhou 310058 P. R. China; ^4^ China Orthopedic Regenerative Medicine Group (CORMed) Hangzhou 310000 P. R. China; ^5^ Department of Sports Medicine Zhejiang University School of Medicine Hangzhou 310000 P. R. China; ^6^ Core Facilities Zhejiang University School of Medicine Hangzhou 310000 P. R. China; ^7^ Dr. Li Dak Sum & Yip Yio Chin Center for Stem Cells and Regenerative Medicine and Department of Orthopedic Surgery of Sir Run Run Shaw Hospital Zhejiang University School of Medicine Hangzhou 310058 P. R. China; ^8^ Qiushi Academy for Advanced Studies Zhejiang University Hangzhou 310058 P. R. China; ^9^ Huzhou Hospital Zhejiang University School of Medicine Huzhou 313000 P. R. China; ^10^ Rehabilitation Department Lishui People's Hospital Lishui 323000 P. R. China

**Keywords:** Amorphous calcium phosphate, Soft‐hard interface, Tissue integration, Tendon‐bone interface, Tendon intrafibrillar mineralization

## Abstract

Soft and hard tissues possess distinct biological properties. Integrating the soft‐hard interface is difficult due to the inherent non‐osteogenesis of soft tissue, especially of anterior cruciate ligament and rotator cuff reconstruction. This property makes it difficult for tendons to be mineralized and integrated with bone in vivo. To overcome this challenge, a biomimetic mineralization strategy is employed to engineer mineralized tendons. The strategy involved infiltrating amorphous calcium phosphate precursors into collagen fibrils, resulting in hydroxyapatite deposition along the *c*‐axis. The mineralized tendon presented characteristics similar to bone tissue and induced osteogenic differentiation of mesenchymal stem cells. Additionally, the interface between the newly formed bone and tendon is serrated, suggesting a superb integration between the two tissues. This strategy allows for biomineralization of tendon collagen and replicating the hallmarks of the bone matrix and extracellular niche, including nanostructure and inherent osteoinductive properties, ultimately facilitating the integration of soft and hard tissues.

## Graphical abstract

A schematic diagram depicting the process of biomimetic mineralized tendon slices fabrication and bone tunnel implantation. The biomimetic mineralization of tendon promotes the tendon‐bone interface integration.

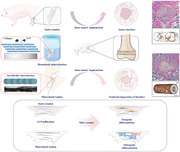

## 1 Introduction

Regenerating the tendon‐bone junction remains a persistent challenge due to its complex hierarchical gradient structure, which comprises the tendon, non‐calcified fibrocartilage, calcified fibrocartilage, and bone tissue.^[^ ^1^ ^]^ The gradient transition of these four components provides for the smooth transmission of forces produced by the muscle to the bone through the tendon, thereby preventing tissue rupture caused by stress concentration.^[^ ^2^ ^]^ However, the intricacy of this natural structure makes self‐healing difficult after an injury. As a result, surgical intervention is often required for patients suffering from tendon‐bone junction rupture.^[^ ^3^ ^]^ Nevertheless, tendons and bones are connected only by fibrous tissue, which cannot integrate them, let alone, form a gradient structure. Thus, re‐rupture after surgery remains a prevalent concern in the clinic.^[^ ^4^ ^]^

Among all tendon‐bone injuries, anterior cruciate ligament (ACL) and rotator cuff injuries are the most severe situations, which disrupt motor function and necessitate surgical intervention. Reconstructive surgery typically involves attaching tendon grafts to the bone by using screws and sutures. Although both tendon and bone are comprised of collagen, the internal of bone collagen is filled with hydroxyapatite (HAP), which significantly enhances its strength and has a greater osteogenic capacity compared to the tendon matrix,^[^ ^5^ ^]^ while the tendon will inhibit the osteogenic differentiation of stem cells and prevent biomineralization of the matrix in vivo.^[^ ^6^ ^]^ As a result, the tendon and bone may integrate poorly even after a prolonged period, possibly leading to both enlargement of the bone tunnel and an increased risk of re‐rupture.

Traditional tissue engineering strategies involve the fabrication of gradient scaffolds in vitro and their transplantation to the injury site to induce the formation of the hierarchical gradient structure in vivo.^[^ ^7^ ^]^ Li et al. constitute a triphasic silk‐based graft in which three regions respectively refer to ligament, cartilage, and bone layers. This triphasic ligamentous graft exhibited the enhancement of osseointegration.^[^ ^8^ ^]^ Kim et al. innovatively fabricated a human collagen‐based multilayer scaffold that emulates the transition from tendon to bone. This scaffold encompasses four distinct layers, each characterized by a unique composition gradient. The initial layer represents the tendon, primarily comprising collagen, followed by an uncalcified fibrocartilage layer composed of collagen and chondroitin sulfate. Subsequently, a calcified fibrocartilage layer, enriched with collagen and a reduced amount of apatite represent the calcified fibrocartilage layer. Finally, the outermost layer serves as bone, exhibiting a composition of collagen interwoven with HAP.^[^ ^9^ ^]^ Among the various scaffold designs for tendon‐to‐bone repair, those incorporating a multiphasic structure comprising both non‐calcified and calcified regions have demonstrated the most promise.^[^ ^10^ ^]^ These biomimetic scaffolds replicate key features of the native tendon‐to‐bone attachment, resulting in improved healing and functional recovery. However, despite advancements in scaffold design and fabrication, the application of these scaffolds remains limited in vivo due to the complex processes and mismatched clinical situations. Throughout the surgical process of tendon‐bone reconstruction, the preservation of tissue integrity is upheld. In this scenario, emphasizing the integration of tendon and bone takes precedence over the mere reconstruction of tendon‐bone structures. This indicated that the gradient strategy was not clinically applicable. Various methods have been explored to enhance tendon‐to‐bone healing, including HAP,^[^ ^11^ ^]^ bone cement,^[^ ^12^ ^]^ bone morphogenetic protein (BMP),^[^ ^13^ ^]^ magnesium,^[^ ^14^ ^]^ and platelet‐derived growth factor.^[^ ^15^ ^]^ These methods have been shown to be effective in accelerating the healing process by increasing new bone or fibrocartilage formation at the interface. However, they are incapable of inducing bone growth into the graft because the osteogenic additives are incompetent in promoting the connection between bone and tendon. This is due to the fundamental differences in the composition of bone and tendon. Collagen fibers are internally mineralized, creating a hybrid structure where HAP is deposited within the fibers in bone. Because of this unique structure, bone has a natural ability to heal completely. If a similar mineralized collagen structure in the tendon can be engineered as in bone, it could be possible to transform tendon‐to‐bone integration into bone‐to‐bone integration, leading to the successful integration of tendon and bone.

To address these challenges, we propose the biomimetic mineralization of tendon collagen to transform soft tissue into hard tissue for enhanced tendon‐bone integration. The interplay between an organic matrix or template and an inorganic mineral phase in biomineralization gives rise to extraordinary properties. Bone, for example, derives its load‐bearing properties from HAP platelets hierarchically deposited in a collagen matrix. In the early stage of biomineralization, the osteoblasts secrete matrix vesicles gathering calcium and phosphate, work as precursors of HAP–amorphous calcium phosphate (ACP)—and finally deposit into collagen. Biomimetic mineralization refers to a strategy driving ACP to infiltrate recombinant collagen and nucleation in the inner space in vitro, which mimics the natural mineralization process in the body.^[^ ^16^ ^]^ The deposition of mineral ions within collagen eventually forms a bionic scaffold that closely resembles the natural bone nanostructure.

**Figure 1:**
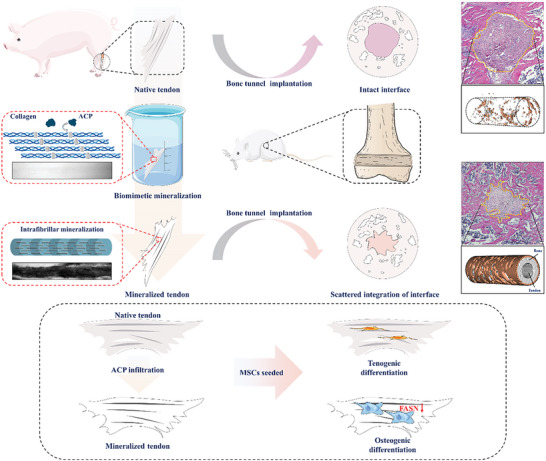
A schematic diagram depicting the process of biomimetic mineralized tendon slices fabrication and bone tunnel implantation.

In this study, tendon tissue was immersed in a biomimetic mineralization system filled with ACP. The ACP precursors infiltrated the collagen fibers of the tendon and sequentially crystallized to realize the tendon collagen biomineralization. This formed an inorganic–organic composite with properties similar to those of native bone. As an osteogenic implant, the mineralized tendon induced osteogenesis of mesenchymal stem cells (MSCs) and narrowed the bone tunnel (**Figure** **1**). This pattern differs from the chaotic deposition of minerals produced by HAP coating or simulated body fluid immersion and significantly improves the mechanical properties of collagen fibers as well as osteogenic induction ability.^[^ ^17^ ^]^ Overall, this study demonstrates a promising approach for the integration of the tendon‐bone interface.

## 2 Results

### 2.1 Characterization of Biomimetic Mineralized Tendon

**Figure 2:**
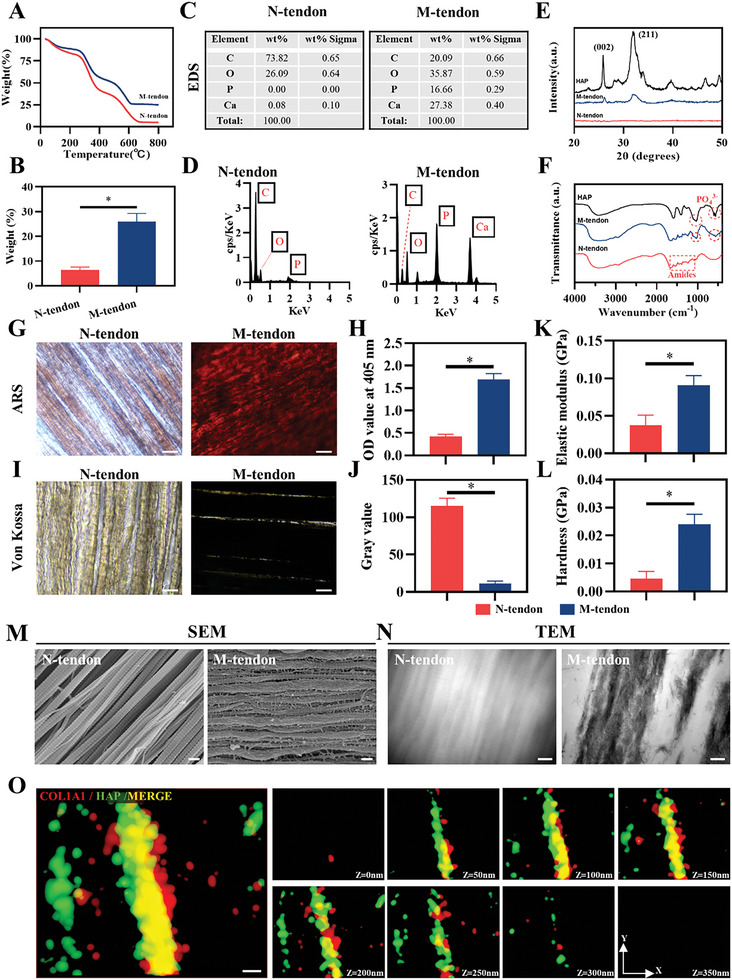
Characteristics of normal and mineralized tendon slices. A) Thermogravimetric analysis (TGA). B) The mineral content of normal and mineralized tendon slices (*n* = 3). C, D) Quantitative analysis of elements. E, F) The chemical and physical characteristics of normal and mineralized tendon slices. G, H) ARS staining and quantitative analysis of normal and mineralized tendon slices. Scale bar = 100 µm. I, J) Von Kossa staining and quantitative analysis of normal and mineralized tendon slices. Scale bar = 100 µm. K,L) The modulus and hardness of normal and mineralized tendon slices *n* = 3. M, N) SEM and TEM of normal and mineralized tendon slices. Scale bar = 200 nm. O) STORM image of the mineralized collagen fibrils suggests that the HAP is in the collagen fibrils. Scale bar = 200 nm.

As the arrangement of tendon collagen fibers was extremely compact, the tendon was sectioned to make ACP infiltrate into the acellular matrix. Then the tendon slices were mineralized for 4 days, as a longer processing time would not bring a significant effect improvement. We performed thermogravimetric analysis (TGA) on normal tendon (N‐tendon) and mineralized tendon (M‐tendon). When the temperature was continuously heated to 800 °C, the mass of the two groups of materials stopped decreasing (**Figure** **2**A). The remaining weight was recorded as inorganic minerals, as the lost weight came mainly from the burning of organic components. The result showed that the minerals accounted for ≈27% of the total weight in M‐tendon while 4% in N‐tendon (Figure 2B). The mineral content of M‐tendon accounted for half the natural bone (50–60%) after 4 days of mineralization. The EDS in X‐ray detection and analysis further proved successful mineralization of the tendon (Figure 2C,D). The peak of calcium and phosphate appeared, and the ratio of these two elements was ≈1.64. X‐ray diffraction was applied to assess the nucleation of HAP within collagen, and characteristic diffraction peaks (002), (211) of HAP appeared after mineralization (Figure 2E). The Fourier transform infrared spectroscopy (FTIR) result showed that tendon collagen had characteristic amide I, II, and III bonds, and the peaks were located at 1655, 1560, and 1240 cm^−1^, respectively. The mineralized tendon not only presented the characteristic groups of collagen but also had the characteristics of HAP. Specifically, the band at 570 cm^−1^ was attributed to the PO_4_^3−^ ν4 bending modes, and the band at 1055 cm^−1^ was attributed to the PO_4_^3−^ ν3 antisymmetric stretching modes. Due to the crystallization of HAP, the former peak was split into two small peaks (Figure 2F). Alizarin red S (ARS) staining and Von Kossa staining showed that the M‐tendon group formed an insoluble dark orange complex with ARS, or replaced silver ions and further reduce silver ions to black metallic silver when exposed to intensive light, while the collagen matrix in normal tendons was little stained with the two dyes (Figure 2G,J). This demonstrated a large amount of calcium deposited within the M‐tendon. The biomineralization process improved the mechanical properties of the tendon dramatically. As nanoindentation testing elucidated, Young's modulus and hardness of the M‐tendon were several times higher than those of the N‐tendon (Figure 2K,L).

Then, the deposition of HAP and the mode of mineralization were observed. Scanning electron microscopy (SEM) results showed that the morphology of the M‐tendon was significantly changed when compared with that of the N‐tendon (Figure 2M). The surface of collagen fibrils was coated by HAP, and the D‐period disappeared. This demonstrated that collagen fibrils induced the depositing of HAP. During preparation for transmission electron microscopy (TEM), the samples were not stained with osmic acid or uranium acetate, so the contrast of collagen fibers of N‐tendon was low in the TEM field, and only the outline of collagen could be seen faintly. In contrast, mineralized collagen was internally deposited with a large amount of HAP, which, with its strong electron scattering ability, formed the dark area. We also noticed that there were blank areas within the field of view of the image, which was evidence of moderate mineralization (Figure 2N). We then used fluorescent dyes to label collagen fibers and calcium, respectively. Through STORM microscope imaging and 3D reconstruction, it was clearly observed that calcium deposition was in the interior of collagen rather than on the collagen surface, indicating the success of intrafibrillar mineralization (Figure 2O). The mineralization depth was further characterized through the energy dispersion spectrum (EDS). Given the scarcity of minerals in normal tendon slices, the feeble signals of calcium and phosphorus pervade the entire field (Figure S1A, Supporting Information). Quantitative analysis reveals a lack of noteworthy mineral augmentation at the cross section (Figure S1B, Supporting Information). Conversely, within the mineralized tendon slices, the signals of calcium and phosphorus gather conspicuously at the crosssection, displaying a noteworthy surge in signal intensity (Figure S1C,D, Supporting Information). These results suggested a full‐thickness mineralization of the matrix.

The above results demonstrated the full‐thickness mineralization of the tendon slice, characterized by intrafibrillar mineralization. The inorganic mineral (hydroxyapatite – HAP) deposits within the organic matrix (collagen) in an orderly manner. This harmonious hybridization of inorganic and organic components significantly enhances the mechanical properties of the matrix.

### 2.2 Biological Properties of Moderately Mineralized Tendon

**Figure 3:**
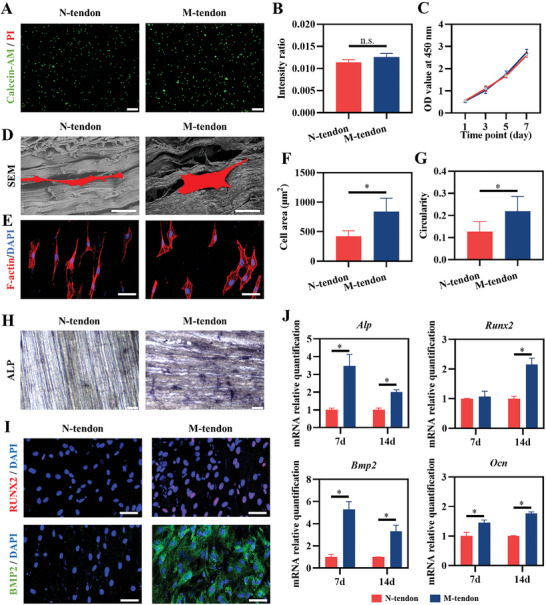
The proliferation and osteogenic differentiation ability of cells on the normal and mineralized tendon slices. A, B) Live/dead cells staining and fluorescence intensity ratio of C3H/10T1/2 after culture on the normal and mineralized tendon slices *n* = 3. Scale bar = 200 µm. C) The proliferation of C3H/10T1/2 measured by CCK‐8 *n* = 6. D, E) The morphology of C3H/10T1/2 was observed under SEM and fluorescence microscope, respectively. Scale bar = 20 µm in (D) and 50 µm in (E). F, G) Cell area and circularity of C3H/10T1/2 *n* = 9. H) ALP staining. Scale bar = 100 nm. I) Immunofluorescent staining of RUNX2 and BMP2. Scale bar = 50 µm. J) mRNA expression levels of osteogenic markers of C3H/10T1/2 culture in the normal and mineralized tendon slices, respectively, n = 3.

Further, we inoculated C3H10T1/2 cells (mouse pluripotent mMSCs line) on the surface of N‐tendon and M‐tendon. After 7 days of culture, dead cells were rarely seen under the fluorescence microscope in either group (**Figure** **3**A). Quantitative analysis was carried out by a fluorescence microplate reader. The ratio of OD535 and OD490 represented the ratio of living cells to dead cells. The results showed no significant difference between the two groups (Figure 3B). At the same time, we used the CCK8 method to track the proliferation of cells for 1, 3, 5, and 7 days. There was no significant difference in the rate of cell proliferation (Figure 3C). These results showed that tendon mineralization did not induce cell death or inhibit cell proliferation.

After the cells were inoculated on the tendon surface for 12 h, the specimens were collected and photographed by SEM with cytoskeleton staining. It was observed that the cell morphology on the surface of the non‐mineralized tendon was slender but was stretched on the surface of the mineralized tendon (Figure 3D,E). Statistical analysis showed that cell area and circularity in the M‐tendon group were larger than in N‐tendon (Figure 3F,G). The cell morphology might be closely related to its differentiation tendency. Therefore, we further induced and detected the osteogenesis ability of C3H cells on the surface of normal tendons and mineralized tendons. After 7 days of culturing with induction, stem cells grown on the mineralized tendon showed stronger ALP activity (Figure 3H). Similarly, immunofluorescence staining results indicated that stem cells on the surface of mineralized tendon expressed higher levels of osteogenic differentiation‐related factors RUNX2 and BMP2 (Figure 3I). Finally, we detected the mRNA expression level of cells. qPCR results showed that mineralized tendon better promoted the expression of Alp and Bmp2 genes in stem cells at 7 days, while the expression of osteogenic‐related genes including Alp, Bmp2, Runx2, and Ocn was increased at 14 days (Figure 3J). In conclusion, M‐tendon exhibited biosafety and displayed the ability to induce osteogenic differentiation of pluripotent MSCs.

#### 2.2.1 Osteogenic Differentiation Mechanism of Mineralized Tendon by Transcriptomic Analysis

**Figure 4:**
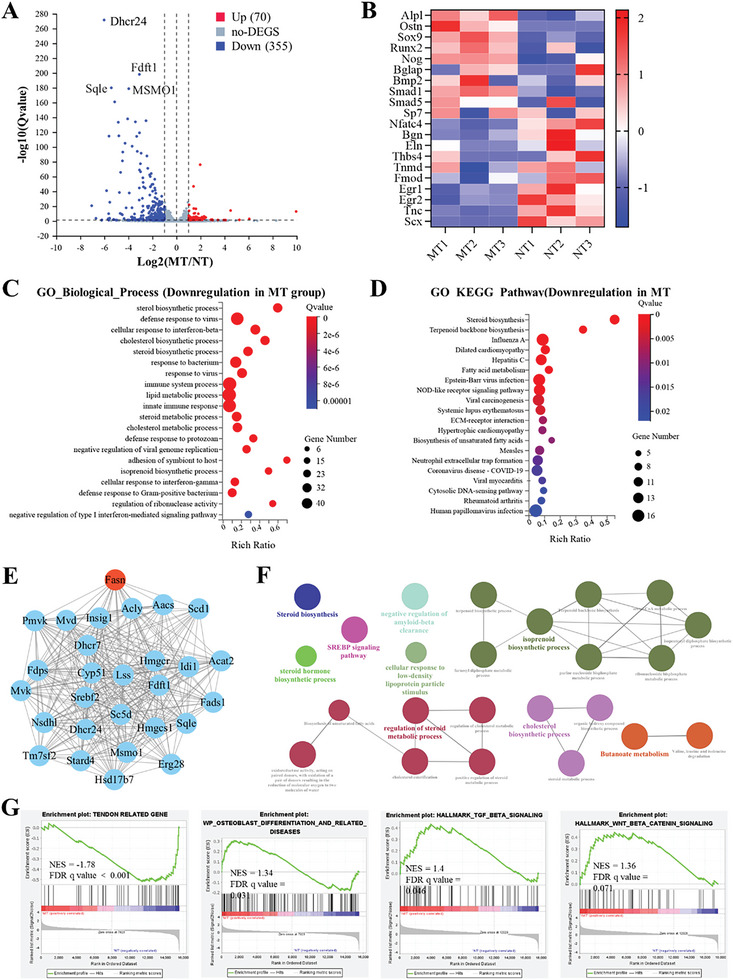
RNA‐seq analysis of the cells cultured on normal and mineralized tendon slices. A) Volcano plot of gene expression (M‐tendon versus N‐tendon; fold change, ≥2; q value < 0.05). B) Heat map of osteogenesis and tenogenesis related genes. C, D) GO analysis of differentially expressed genes. E) Protein–protein interaction network diagram of differentially expressed genes. F) Enrichment analysis of (E). G) GSEA analysis of all genes in M‐tendon and N‐tendon groups. NES, normalized enrichment score; FDR, false discovery rate.

The comprehensive role of M‐tendon and N‐tendon in mediating cell behavior was then analyzed by conducting RNA‐sequencing (RNA‐seq) studies on the cultured cells (**Figure** **4**). The gene sets of mice were employed as a reference. We found 355 genes downregulated and 70 genes upregulated in comparing M‐tendon with N‐tendon (fold change ≥2; q value <0.05) (Figure 4A). When comparing gene expression related to osteogenic differentiation and tendon differentiation, we observed that genes including Alpl, Ostn, Sox9, Runx2, Nog, Bglap, Bmp2, Smad1, Smad5, and Sp7 were highly expressed in the M‐tendon group, while genes including Nfatc4, Bgn, Eln, Thbs4, Tnmd, Fmod, Egr1, Egr2, Tnc, and Scx were mainly expressed in the N‐tendon group (Figure 4B). Gene Ontology (GO) analysis revealed that the expression level of the biological process and signaling pathway related to lipid metabolism was significantly downregulated in the M‐tendon group (Figure 4C,D). We thought that this was because the activation of lipid metabolism required a soft matrix, so there would be corresponding down‐regulation after tendon mineralization. In the next step, we investigated the interactions between proteins by analyzing the key subnetwork, which includes genes like Dhcr24 and fdft1 (Figure 4E). It was conducted further enrichment analysis of the genes in this key subnetwork and found that they were also associated with lipid metabolism (Figure 4F). Within this network, fatty acid synthase (FASN) had high connectivity within the module (Figure 4E). Our literature search revealed that some studies have reported that the downregulation of FASN could inhibit the lipogenic differentiation of stem cells and enhance their osteogenic differentiation potential.^[^ ^18^ ^]^ FASN serves as a pivotal enzyme in the intricate process of de novo biosynthesis of fatty acids and is expressed in adipocytes and osteoblasts. Notably, FASN participates in the crosstalk between adipose and bone tissue metabolism.^[^ ^19^ ^]^ FASN was proved to be up‐regulated during adipogenic differentiation of MSCs.^[^ ^20^ ^]^ In a study on the regulation of MSCs osteogenic differentiation, betaine showed a significant down‐regulation effect on genes associated with lipogenesis such as PPARγ, CEBPα, and FASN.^[^ ^21^ ^]^ The TGF‐β signaling pathway in osteoclastogenesis and immune dysregulation, as well as the anomalous accumulation of FASN in Langerhans cells, have been proven to be associated with osteolytic Langerhans cell histiocytosis.^[^ ^22^ ^]^ Further studies on FASN hold the potential to facilitate the osteogenic differentiation of stem cells and promote bone tissue regeneration. This finding supports our conjectural hypothesis that the mineralization of tendons could promote the osteogenic differentiation of stem cells. Because of the inherent limitations of differentially expressed gene analysis, we performed gene set enrichment analysis (GSEA) on all genes. The results showed that tendon‐related genes were downregulated in the mineralized tendon group while osteoblast differentiation genes were upregulated. Specifically, the TGF‐β signaling pathway and the WNT‐β‐catenin signaling pathway were both notably upregulated (Figure 4G). TGF‐β and WNT‐β‐catenin signaling are recognized as crucial regulators of osteogenic differentiation in bone regeneration. Overall, these in vitro findings demonstrated that mineralized tendon induced the osteogenesis of cells by promoting the expression of osteogenic genes and initiated related signaling pathways.

### 2.3 Mineralized Tendon Promotes Tendon‐Bone Regeneration through Tissue Fusion

**Figure 5:**
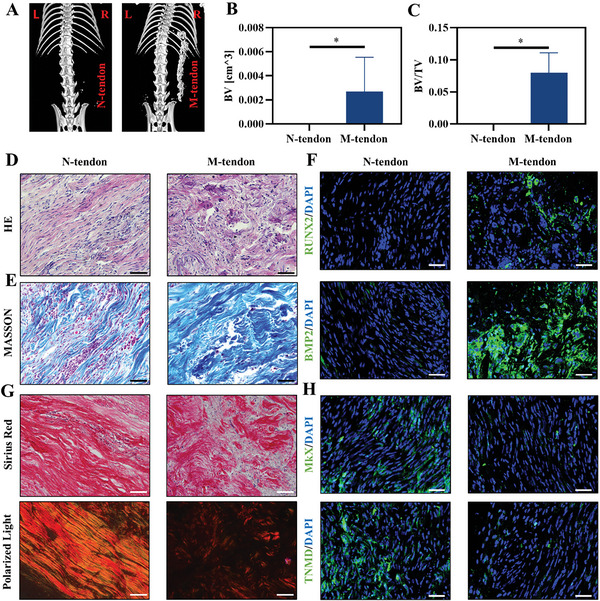
Biocompatibility and ectopic ossification of normal and mineralized tendon slices in vivo. A–C) Representative micro‐CT images and quantitative analysis of results. D,E) HE and MASSON staining of samples. Scale bar = 50 µm. F) Immunofluorescent staining of RUNX2 and BMP2. Scale bar = 50 µm. G) Sirius Red staining imaged under polarization microscopy. Scale bar = 50 µm. H) Immunofluorescent staining of MKX and TNMD. Scale bar = 50 µm.

To test the osteogenesis ability of M‐tendon in vivo, we curled the tendon slice into a strip and embedded it subcutaneously in the mouse. After 4 weeks, micro‐CT results showed that there was a strip of high signal shadow on the right side of the spine in the experimental group, while there was no signal at the same position in the control group (**Figure** **5**A). The bone volume and bone volume fraction of the experimental group were much higher than those of the control group (Figure 5B,C). The staining of tissue sections, including HE and MASSON, showed that the matrix arranged in the control group was more parallel and orderly (Figure 5D,E). The sample from the N‐tendon group expressed little osteogenic differentiation‐related proteins BMP2 and RUNX2. In the M‐tendon group, BMP2 and RUNX2 were significantly upregulated, and the content of the collagen matrix was a bit like the bone matrix (Figure 5F). Meanwhile, more positive areas of MKX and TNMD were observed, which suggested a stronger tenogenic differentiation property (Figure 5H). Sirius Red staining demonstrated that the specimens from the M‐tendon group had poorly organized tissue and collagen fiber alignment as compared with the control specimens. This indicated a transformation of bone‐like tissue (Figure 5G). The tartrate‐resistant acid phosphatase (TRAP) staining revealed recruitment of osteoclasts (Figure S2, Supporting Information). These results proved that mineralized tendons had the characteristics of bone induction.

**Figure 6:**
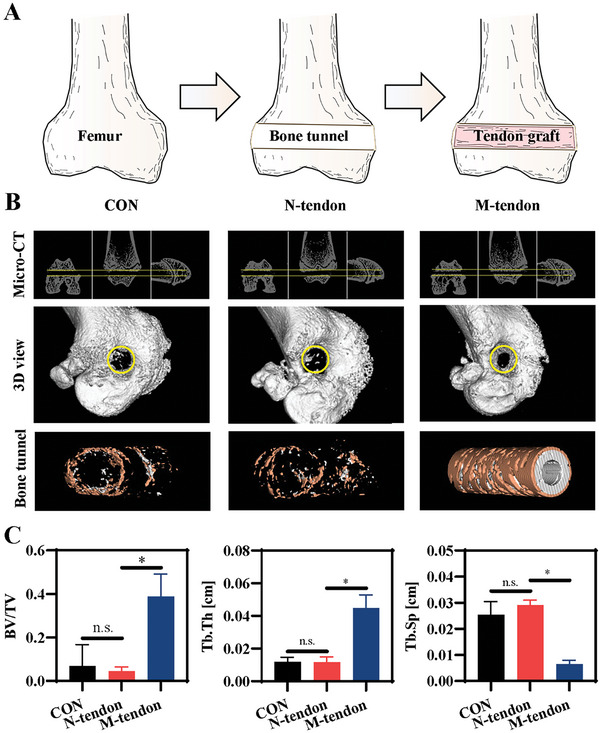
Micro‐CT analysis after graft implantation in a tendon‐bone integration model. A) Scheme of operation procedure. B) The horizontal section, coronal section and vertical section bone tunnels were measured, and the 3D images were reconstructed. C) Quantitative analysis of neo‐bone in bone tunnel zone.

To further investigate the bone induction property and potential clinical application, we established a femoral condyle drilling model of mice (**Figure** **6**A). The normal or mineralized tendon slice was rolled into a round strip and passed through the bone tunnel. After 4 weeks, micro‐CT imaging analysis showed that the bone tunnel in the M‐tendon group was significantly narrowed, while the bone tunnel size in the N‐tendon group was little changed (Figure 6B). The newly formed bone mass was evaluated by analyzing the bone volume/total volume (BV/TV) fraction. The mineralized tendon induced formation around the implant with a significantly higher bone mass, and good integration of neo‐bone (orange area) and implants (grey area) was observed (Figure 6B,C). To characterized the spatial form of bone trabecular, trabecular thickness (Tb.Th) and trabecular space (Tb.Sp) were analyzed in the bone tunnel of the three groups. Tb.Th in the M‐tendon group showed the highest value after 4 weeks of implantation, and decreased trabecular spacing implied a more complete bone structure (Figure 6C). Furthermore, higher values of Tb.Th and the lower values of Tb.Sp indicated more active bone anabolism as opposed to catabolism.

**Figure 7:**
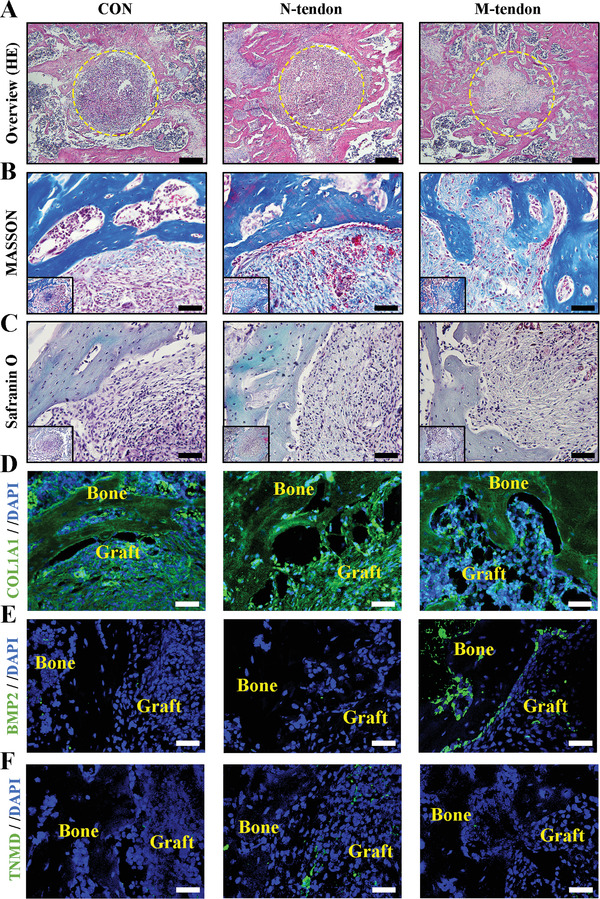
Histological analysis of the tendon‐bone integration process. A–C) HE, MASSON, and Safranin O staining of bone tunnel section after 4 weeks of implantation. Scale bar = 200 µm in (A) Scale bar = 40 µm in (B,C). D–F) Immunofluorescent staining of COL1A1, BMP2, and TNMD in the control, normal and mineralized tendon groups after surgery. Scale bar = 50 µm.

The histological results showed the bone tunnel was mainly filled with fibroblasts and blood cells as no graft was implanted in the control group. The disordered tissue was organized and replaced by fibrous tissue gradually. In the N‐tendon group and M‐tendon group, the bone tunnel was filled with normal tendons or mineralized tendons, the collagen matrix of grafts could be stained with aniline blue in MASSON staining (**Figure** **7**B). In particular, the diameter of the bone tunnel in the control and N‐tendon groups did not dwindle and the interface between the tendon tissue and the native bone tissue was arc‐shaped. Conversely, the soft tissue at the center of the mineralized tendon was significantly reduced, and the soft tissue was intertwined with the neo‐bone (Figure 7A,B,D). This zigzag shape helped increase the contact area between bone and tendon tissue, so as to improve the adhesion of the interface. The mineralized tendon in contact with bone has been initiating the remolding and transforming it into bone. Potential osteogenesis was assessed by IF staining of BMP2 (green) and tenogenesis by TNMD (green). Significant positive expression of BMP2 was observed in mineralized tendons, which was conducive to new bone formation (Figure 7E). Accompanied by decreased staining of tendon induction marker (TNMD, Figure 7F), it can be speculated that the biological characteristics of the tendon had become bone‐like after mineralization. Similar to the findings from subcutaneous implantation, the presence of osteoclasts was identified within the bone tunnel grafts (Figure S3, Supporting Information). The mineralized tendon slices effectively attract and engage osteoclasts, thereby initiating the intricate process of bone remodeling and formation. Furthermore, typical cartilage features were not detected by Safranin O staining at the interface (Figure 7C). The results suggested that the M‐tendon transformed into bone tissue through intramembrane osteogenesis directly instead of through endochondral osteogenesis. In conclusion, the femoral tunnel drilling model proved that M‐tendon retained the characteristics of soft tissue (the areas not in contact with the primary bone tissue in the middle), but it could transform into bone tissue and form a perfect integration with the primary bone tissue.

## 3 Summary and Discussion

This study aimed to develop a mineralized tendon implant via ACP infiltration in a biomimetic mineralization system. The mineralized tendon induced osteogenesis of MSCs and generated a serrated interface in vivo. This strategy facilitated neo‐bone growth across the interface and facilitated the integration of the tendon‐bone interface.

The process of healing tendons and ligaments to bones presents a significant challenge in clinic due to the intricate structure, composition, cell population, and mechanics of the interface.^[^ ^23^ ^]^ Tissue engineering has made remarkable progress, and various approaches, such as advanced biomaterials, bioactive growth factors, and multiple stem cell lineages have been developed to facilitate healing at this tissue interface. Biomaterials played the most important role in promoting tissue regeneration, they provided structural support and appropriate biomechanical and biochemical properties that emulate the natural microenvironment.

Currently, a common strategy involves utilizing multilayer biomaterial scaffolds to replace the tendon‐bone interface. However, this strategy has yielded limited success in research due to challenges in achieving a continuous functionalized gradient and dispersing the mechanics properly. The interface between the two components is affected by stress concentrations, and the technical requirements remain impractical for clinical applications. Therefore, the application of structural bionics strategies remains a significant challenge. The absence of hierarchical structural regeneration processes underscores the urgency for novel approaches to enhance functional integration and promote the regeneration of tendon‐bone structures. In cases where the scaffold fails to achieve continuous gradient construction in vivo, it is essential to assess the necessity of a multilayer scaffold that encourages either bone or tendon formation. During the surgical reconstruction of the rotator cuff and ACL, the presence of native tendon and bone tissue at the interface raises the critical issue of whether the transitional side can generate sufficient bonding to unite the two tissues.

The process of bone ingrowth plays a pivotal role in achieving integration between the graft and the bone tunnel. By controlling the composition, structure, and morphology of the gradient scaffold biomaterial, it is possible to influence cell attachment, proliferation, and differentiation to achieve optimal tendon‐bone interface repair. Upon culturing bone marrow‐derived mesenchymal stem cells on a scaffold featuring a mineral gradient, it was observed that cells contacted with a matrix exhibiting a higher mineral content demonstrated earlier manifestation of osteogenic behavior compared to those on an unmineralized matrix.^[^ ^24^ ^]^ Polyethylene terephthalate (PET) exhibits commendable mechanical properties, rendering it a viable choice for employment as an artificial ligament in ACL reconstruction. Nevertheless, PET encounters challenges with tissue ingrowth leading to a relatively high rate of graft failure in the long‐term follow‐up. Calcium phosphate coating on the PET surface via electrodeposition enhanced the graft‐bone integration.^[^ ^25^ ^]^ Biological scaffolds can be composed of biodegradable or synthetic materials and can have different shapes and properties to suit a variety of repair needs. The combination of biodegradable and non‐biodegradable scaffolds provides space for bone tissue ingrowth, while the release of BMP‐7 exerts additional impetus toward osteogenesis.^[^ ^26^ ^]^

Implant‐induced bone tissue ingrowth is an effective strategy to promote interfacial integration.^[^ ^27^ ^]^ Several studies have modified nanofiber scaffolds through spatial control of mineral content to not only mimic changes in minerals but also create mechanical gradients.^[^ ^28^ ^]^ The incorporation of minerals facilitates bone formation and narrows the bone channels, yet the newly formed bone fails to integrate into the implant and establish a solid bond because the minerals and implant were merely attached or mixed physically and performed their respective functions. The minerals induce bone formation and eventually catabolize.^[^ ^29^ ^]^ If the minerals and implant can be organically combined, it would be possible to avoid the aforementioned problems.

Biomimetic mineralization technology has demonstrated the ability to create organic/inorganic hybrids by engineering minerals and collagen. Previous studies have employed the biomimetic mineralization strategy for biomimetic bone graft fabrication.^[^ ^30^ ^]^ In simulating the bone formation process, ACP is synthesized in vitro and then crystallized within the collagen, extending along the *c*‐axis that is parallel to the long axis of collagen to form mineralized collagen.^[^ ^31^ ^]^ Mineralized collagen is the primary composite of bone matrix. This makes an effective integration with native bone while simultaneously inducing bone formation in vivo. Nevertheless, recombinant collagen lacks sufficient mechanical properties to replace the function of the tendon. The biomimetic mineralization of native tissues on a micrometer scale is still not documented, although the scale of ACP is theoretically tiny enough to enter into the interspace of tissue fibrils. As tendons have the same collagen type as bone (type I), mineralization of tendon tissue via biomimetic mineralization is effective in establishing a firm bond at the tendon‐bone interface. The tiny ACP permits its infiltration into the collagen surrounding the tendon surface. Although ACP nucleation occurs prior to its migration to the inner regions of the tendon tissue, the mineralized layer is sufficient to form a tightly bound interface with neo‐bone. Within the mineralized layer of the tendon, mineralized collagen fibers exhibit nanoscale intrafibrillar oriented minerals, akin to the bone, which stimulate bone tissue growth while retaining the tendon's mechanical properties.

The stiffness of the extracellular matrix and the presence of calcium and phosphate ions have long been known to influence stem cell differentiation. The osteogenic properties of calcium and phosphate scaffolds are attributed to their ability to regulate extracellular ion calcium and phosphorus concentrations in stem cells through Ras/Raf/Erk‐dependent signaling pathways or ATP‐adenosine controlled mechanisms. However, the addition of calcium and phosphate alone has a limited ability to regulate the expression of osteogenesis‐related genes (RUNX2, OCN, PDPN, and DMP1). In contrast, biomimetic intrafibrillar mineralization of collagen by HAP significantly upregulates osteogenesis‐related genes in bone marrow MSCs.^[^ ^30a^ ^]^ In this study, mineralized tendons promoted the expression of osteogenic genes and initiated related signaling pathways, which suggests that intrafibrillar mineralization collagen may be necessary to influence bone marrow MSC differentiation in the absence of osteoinductive factors.

Furthermore, the remarkable osteogenic potential of mineralized tendons implies that biomimetic mineralization‐based implants could be viably employed in the context of bone regeneration therapy. Collagen membranes featuring intrafibrillar mineralization demonstrated exceptional efficacy in terms of osteoinduction and osteoconduction, leading to successful instances of ectopic bone formation within living organisms.^[^ ^32^ ^]^ Notably, mineralized collagen scaffolds constructed by nucleic acid‐based biomimetic mineralization systems have been substantiated as highly effective in bone tissue engineering and regenerative medicine.^[^ ^30b^ ^]^

In conclusion, autologous soft tissues represent the quintessential reservoir for tissue regeneration, while ACL and rotator cuff reconstruction are challenging in clinic, because of the poor tendon‐bone interface integration. This study proposed a strategy to mineralize the tendon collagen resulting in a bone affinitive interface. Mineralized tendon with osteal physicochemical and biological properties facilitates the tendon‐bone interface integration, although the mineralization degree is less than bone. It would be promising to optimize the biomimetic mineralization system to improve the mineralization degree and efficiency of soft tissue in the future. Nonetheless, we believe our strategy provides an alternative procedure for ACL and rotator cuff reconstruction and bone regeneration, which would eventually promote clinical outcomes.

## 4 Experimental Section

### Preparation of Decellularized Tendon Slices

The acellular tendon slices were acquired via a freeze‐thaw procedure. Briefly, tendons were harvested from swine forelimbs, and aponeuroses were removed. Then, the tendon was cut into 1.5 cm × 1.5 cm pieces. The samples were subjected to liquid nitrogen and phosphate buffer saline (PBS) at 37 °C for a total of ten cycles. Next, the samples were immersed into 1% (v/v) sodium dodecyl sulphate for 1 h and rinsed with PBS. The decellularized tendons were embedded in an optimal cutting temperature compound, and 80µm thick slices were obtained using a freezing microtome (Leica CM1950, Leica, Wetzlar, Germany). The slices were washed thrice with PBS and cross‐linked with 0.3 m 1‐(3‐dimethylaminopropyl)−3‐ethylcarbodiimide hydrochloride/0.06 m 1‐(3‐dimethylaminopropyl)−3‐ethylcarbodiimide hydrochloride for 4 h.

### Biomineralization of Decellularized Tendon Slices

The mineralizing solution was prepared by mixing equal volumes of solution A [3.33 mm CaCl_2_, 300 mm NaCl, 480 µg mL^–1^ poly‐aspartic acid (Mw = 8000–11 000 kDa)] and solution B (19 mm NaH_2_PO_4_). Decellularized tendon slices were immersed into more than ten times their volume of mineralizing solution and incubated at 37 °C for a total of 4 days. The mineralizing solution was refreshed every 12 h to facilitate the mineralization process.

### Scanning Electron Microscopy and Energy Dispersion Spectrum

The specimens were fixed with 2.5% glutaraldehyde, then dehydrated with an ascending ethanol series (30–100%), critical‐point dried (Hitachi, HCP‐2), and sputter‐coated with gold. The morphology and element composition of tendon slices were recorded via scanning electron microscopy (SEM, Hitachi S‐8010, Japan) at 3 kV and energy dispersion spectrum (EDS) at 15 kV, respectively. To characterize the depth of mineralization, the elemental distribution in the cross section was detected via EDS.

### Transmission Electron Microscopy

The specimens were fixed with 2.5% glutaraldehyde, dehydrated with an ascending ethanol series (30–100%), embedded in epoxy resin, sectioned to 80 nm thickness using a Leica ultramicrotome (Leica EM UC7), and collected on copper grids. Without staining with uranium acetate, the ultrastructure of specimens was observed under transmission electron microscopy (TEM, Hitachi, H‐7650) at 120 kV.

### Thermogravimetric Analysis (TGA)

TGA was performed with a thermal gravimetric analyzer (DSC1/400, TGA/DSC1 1100SF‐MS, METTLER‐TOLEDO, Switzerland) after specimens were dried in a drying oven at 65 °C overnight. The specimen was heated from 25 to 200 °C for dehydration and 200 to 600 °C for organic decomposition. The ratio of the final weight and weight loss at 800 °C was analyzed.

### X‐Ray Diffraction (XRD) and Fourier Transform Infrared Spectroscopy (FTIR)

The crystal structure of specimens and HAP powder was characterized by XRD (D8 Advance, Bruker, Germany) operated at 45 kV and 40 mA. All the specimens were air‐dried at 65 °C overnight and pulverized to produce fine powders. The recorded patterns were analyzed using JADE6 software (Materials Data Incorporated). FTIR (Nicolet iN10, Thermo Scientific, USA) was performed to assess their chemical composition. The specimens were dried and pulverized as above‐mentioned. After being mixed with potassium bromide, the specimens were analyzed with FTIR (Iraffinity‐1, Shimadzu, Japan).

### Mechanical Properties Assay

Nanoindentation tests were performed utilizing a Nano‐indenter (G200, Agilent Tech.) to characterize the mechanical properties of specimens. The quantitative data was calculated based on the standard mathematical model employed by the apparatus.

### Alizarin Red S Staining and Von Kossa Staining

Alizarin red S staining (ARS) and Von Kossa were used to identify the mineral composition. The tendon slices were rinsed with distilled deionized water and incubated in 2% ARS solution for 30 min. For Von Kossa staining, the specimens were incubated with 5% silver nitrate solution for 15 min under ultraviolet. Then, a 5% sodium thiosulfate solution was employed to remove the residual silver nitrate. The stained specimens were observed and photographed using microscopy.

### 3D‐STORM Imaging

The mineralized tendon slices were labeled with a fluorescent reagent by immunofluorescence staining. The slices were first fixed with 4% paraformaldehyde. After washing thrice, the samples were blocked with 1% bovine serum albumin (BSA) for 1 h at room temperature and then overnight at 4 °C with primary anti‐collagen I (ab34710). Then, the samples were labeled with secondary antibodies for 1 h and calcein (Aladdin, C118587) for 30 min. The samples were rinsed thoroughly and subsequently immersed in the imaging buffer before STORM imaging. All STORM imaging data were acquired by virtue of a Nikon Ti‐E inverted microscope (N‐STORM/A1R, Nikon, Japan) and analyzed by Nikon NIS‐Elements AR software.

### Cell Culture

MSCs lines (C3H/10T1/2, The Chinese Academy of Sciences, Shanghai) were cultured in a growth medium (L‐DMEM supplemented with 10% FBS and 1% penicillin/streptomycin). Cell culture was performed at 37 °C and 5% CO_2_ in a humidified environment. The medium was renewed every 3 days. After the designated culture time, the cells were used for further study.

### Cell Viability Assay

Prior to the experiments, the slices of both normal and mineralized tendons underwent a thorough cleansing process. They were subjected to three successive washes with 75% alcohol, lasting 30 min each, in order to achieve sterilization and eliminate the residues. Subsequently, the sterilized slices were further washed three times with PBS, with each wash lasting 15 min, to thoroughly remove the remnants of the 75% alcohol. The normal and mineralized tendon slices were then placed in each well, respectively, before C3H cells were seeded in 98‐well plates at a concentration of 1 × 10^3^ cells per well and cultured for 1, 3, 5, and 7 days. The medium was replaced with 10% CCK‐8 (Cell Counting Kit‐8, Dojindo, CK04) solution at the designated time and incubated for 1 h. Cell viability was quantified by a microplate reader (Varioskan Flash, Thermo Fisher Scientific, USA) at a wavelength of 450 nm.

### Live/Dead Cell Staining

After cultured for 7 days on mineralized slices, cells were centrifuged, rinsed with 1 × assay buffer, and resuspended with a density of 1×10^5^‐1×10^6^ cells mL^–1^. Measured 200 µL of cell suspension was mixed with 100 µL of staining solution (2 µm calcein‐AM and 4.5 µm propidium iodide) and incubated at 37 °C for 15 min. Live and dead cells were visualized with confocal laser scanning microscopy. Their fluorescence intensity was quantified by a microplate reader (Thermo Scientific 5 250 040) at 490 nm and 545 nm, respectively.

### Cell Morphology

For immunofluorescent staining, specimens were fixed with 4% paraformaldehyde and permeabilized using 0.1% Triton X‐100. The samples were further blocked using 1% BSA in PBS for 1 h and stained with Alexa Fluor 488‐conjugated phalloidin as well as 4′,6‐diamidino‐2‐phenylindole. The sample preparation for SEM is described above.

### Osteogenesis Differentiation

The C3H cells were seeded on mineralized slices at a density of 5000 cells cm^–2^. After 3 days, the growth medium was replaced with the osteogenic medium (growth medium supplemented with 10 mm β‐glycerophosphate, 10 nm dexamethasone, 50 µg mL^–1^ ascorbic acid) to induce the formation of calcium nodules. The osteogenic medium was replaced every 3 days. Specimens were harvested for further testing at designated induction times.

### Quantitative Real‐Time Polymerase Chain Reaction (qPCR) Analysis

After cells were cultured in osteogenic medium for 7 and 14 days, quantitative real‐time PCR (qPCR) analysis was performed to evaluate the expression level of osteogenesis‐related genes. Total RNA was extracted using an RNA extraction reagent (Takara, 9109) according to the protocol. Thereafter, cDNA was synthesized and amplified in a reaction system. The qPCR was performed with a SYBR Green Mix (Takara, RR420A) in a fluorescence signal detection device (Roche, LightCycler 480).

The primers used were the following:

**Table:** 

Genes	5′‐3′	Sequence
Mouse *Alp*	Forward Reverse	ATCGACGTGATCATGGGTGG GAGAGCGAAGGGTCAGTCAG
Mouse *Runx2*	Forward Reverse	AATTGCAGGCTTCGTGGTTGAG GCTGTATGGTGAGGCTGGTAGG
Mouse Bmp2	Forward Reverse	GATCACCTCTCTTCCTCAGCCC CAACACTAGAAGACAGCGGGT
Mouse Ocn	Forward Reverse	TGAAGACCGCCTACAAACGC ACAGGGAGGATCAAGTCCCG

### Transcriptome Sequencing and Data Analysis

C3H cells were cultured on the surface of N‐tendon or M‐tendon for 14 days, and then the cells were collected to extract RNA. After verification of its integrity, the extracted RNA was reverse‐transcribed to create a cDNA library for subsequent sequencing. Gene ontology (GO) and KEGG pathway enrichment analysis were performed on the data analysis platform provided by BGI (biosys.bgi.com). The protein–protein interaction network map and its subsequent gene enrichment analysis were produced in string‐db.org and by Cytoscape software. To be specific, the genes downregulated in the M‐tendon group were first subjected to protein‐protein interaction prediction analysis. Subsequently, the resultant files were input into Cytoscape, after that the key subnetwork was obtained by utilizing the MCODE plugin's functionality. Finally, the genes encompassed within the subnetwork were subjected to enrichment analysis.

### Animal Experiments

Animal experiments were approved by the Zhejiang University Experimental Animal Welfare and Ethics Committee under Institutional Animal Care and Use Committee guidelines (ZJU20210029). Eight‐week‐old mice were obtained from a licensed vendor and reared at an environmental temperature of 25 ± 2 °C with a dark/light cycle of 12 h. The animals were acclimated for 7 days before experiments. All operations abided by standard surgical procedures.

### In Vivo Biocompatibility and Ectopic Osteogenesis Ability

Ten mice were randomly assigned into a normal tendon group or a mineralized tendon group (five replicates for each group). Freshly prepared specimens were sterilized with 75% ethanol before subcutaneous implantation. Briefly, mice were anesthetized with pentobarbital. In the implantation, the surgical area was prepared and a 6 mm longitudinal incision was made at the middle of the dorsal skin. Muscle and skin were separated with scissors to create a subcutaneous pocket. The acellular tendon slice or mineralized acellular tendon slice was rolled into a cylinder with a diameter of 0.8 mm and then implanted into the subcutaneous pocket. Each specimen was sutured 5 mm away from the incision. After implantation, the incision was sutured, and the mice were monitored until recovery from anesthesia.

### Bone Tunnel Drilling and Transplantation Model

Control, acellular tendon, and mineralized acellular tendon groups were randomly assigned (five mice for each group). Sterilized grafts were passed through 0.8 mm bone tunnels in the distal femur. Briefly, mice were anesthetized with pentobarbital. A ≈5 mm incision was created in the medial of the distal femur. All the mice underwent medial arthrotomy and patella dislocation to expose the distal femur. A bone tunnel ≈4 mm in depth and 0.8 mm in diameter was drilled inside‐out in the femoral metaphysis by using a dental drill. The bone tunnel was irrigated with normal saline to remove bone debris and subsequently filled according to the designated group. Muscle, fascia, and skin were sutured in turn after repositioning the patellar.

### Micro‐Computed Tomography Imaging and Analysis

After 4 weeks of implantation, the mice were sacrificed for imageology. Scans were performed at a voltage of 80 kV and a resolution of 18 µm per pixel using micro‐computed tomography (micro‐CT, U‐CT‐XUHR, Milabs, Netherlands). The pixels exhibiting a CT value >1400 were designated as bone. The images were reconstructed and analyzed via Imalytics Preclinical.

### Histology, Immunohistochemistry, and Immunofluorescence Assays

The mice of each group were sacrificed at 4 weeks post‐implantation. Implants or distal femurs containing the bone tunnel were harvested and fixed with 4% formaldehyde. Samples were decalcified with 10% ethylene diamine tetra‐acetic acid thoroughly, dehydrated through an ascending graded series of ethanol (50–100%), and embedded in paraffin. The embedded samples were sectioned longitudinally at a thickness of 7 µm for histological examination. The sections were stained with hematoxylin & eosin (HE), Safranin O, and MASSON staining.

For immunofluorescence and immunohistochemistry analysis, the staining procedure included deparaffinization, hydration, antigen retrieval, and antibody incubation. Endogenous peroxidase inactivation and diaminobenzidine treatment were acquired for immunohistochemistry analysis. The following antibodies were used in the study: anti‐BMP2 (Beyotime, AF0075), anti‐RUNX2 (Sigma, HPA022040), anti‐COL1 (Abcam, ab34710), anti‐COL2 (Santa Cruz, sc‐52658), and anti‐TRAP (Santa Cruz, sc‐376875). The stained sections were examined under a confocal fluorescence microscope (Nikon A1R, Japan) or an optical microscope.

### Statistical Analysis

Quantitative data are presented as mean ± standard deviation (SD). Student's t‐test was performed to assess whether statistical differences existed between two groups. Multiple comparisons were performed with a one‐way analysis of variance (ANOVA) and Tukey's post‐test. P values of <0.05 were considered statistically significant. The significance level is presented as ^*^ *p* < 0.05.

## Conflict of Interest

The authors declare no conflict of interest.

## Author Contributions

Y.C., Y.Z., and X.C. contributed equally to this work. Y.C. and X.C. designed the project. X.C., W.S., and Z.Y. supervised the project. Y.C., Y.Z., and X.C. performed the experiments and analyzed the data. Y.C. and Y.Z. wrote the manuscript. Y.C., Y.Z., and X.L. revised the manuscript. J.H., B.Z., T.Z., and C.F. conduct animal experiments. W.Y. supervised the 3D‐storm imaging. H.P. supervised the biomimetic mineralization procedure. All authors discussed and approved the results and manuscript.

## Supporting information

Supporting Information

## Data Availability

The data that support the findings of this study are available from the corresponding author upon reasonable request.
