# Supplementary material for: Biomimetic Intrafibrillar Mineralization of Native Tendon for Soft–Hard Interface Integration by Infiltration of Amorphous Calcium Phosphate Precursors
Source: Adv Sci (Weinh). 2023 Oct 23;10(34):2304216. doi: 10.1002/advs.202304216 (PMC10700236; doi:10.1002/advs.202304216)
Supplement: Supplementary file 1 — Supporting Information [file ADVS-10-2304216-s001.pdf]

ADVANCED SCIENCE
Open Access

Supporting Information

for *Adv. Sci.*, DOI 10.1002/advs.202304216

Biomimetic Intrafibrillar Mineralization of Native Tendon for Soft–Hard Interface Integration by Infiltration of Amorphous Calcium Phosphate Precursors

Yangwu Chen, Yuxiang Zhang, Xiaoyi Chen, Jiayun Huang, Bo Zhou, Tao Zhang, Wei Yin, Cailian Fang, Zi Yin, Haihua Pan, Xiongfeng Li, Weiliang Shen and Xiao Chen**

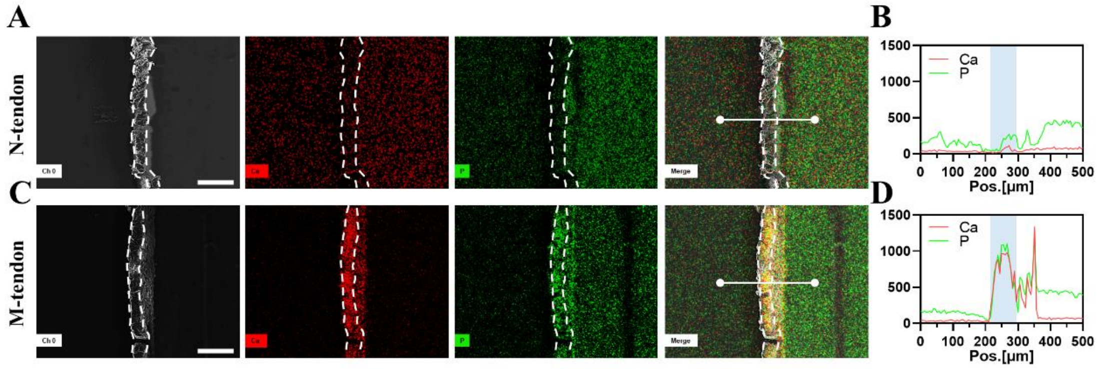

Figure S1 The CaP distribution of cross section (white dashed line and blue region) in N-tendon and M-tendon. (A) The corresponding element mapping of N-tendon. (B) Element distribution in the line scanning crossing the section of N-tendon (solid white line). (C) The corresponding element mapping of M-tendon. (D) Element distribution in the line scanning crossing the section of M-tendon (solid white line). Scale bar = 200 nm.

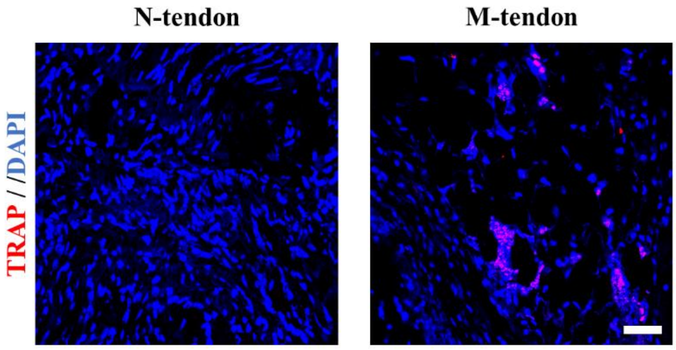

Figure S2 Immunohistochemical staining of TRAP confirmed the participation of osteoclast in the ectopic osteogenesis process.

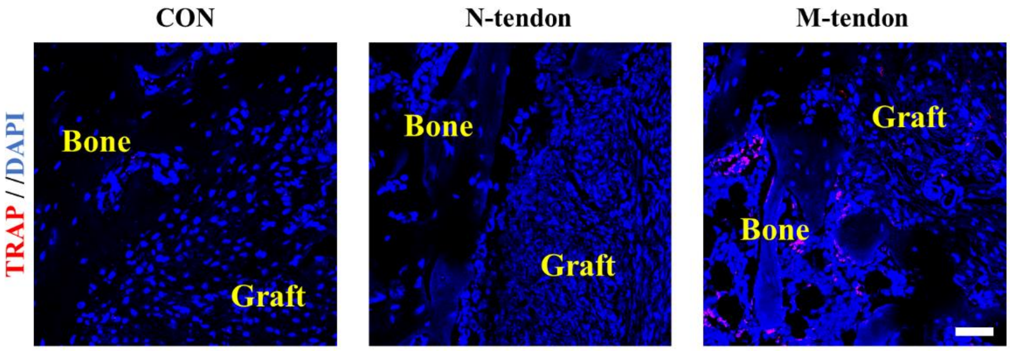

Figure S3 Immunohistochemical staining of TRAP confirmed the participation of osteoclast in the bone-tendon integration process.
